# Efficient Regioselective Synthesis of Novel Water-Soluble 2*H*,3*H*-[1,4]thiazino[2,3,4-*ij*]quinolin-4-ium Derivatives by Annulation Reactions of 8-quinolinesulfenyl Halides

**DOI:** 10.3390/molecules26041116

**Published:** 2021-02-20

**Authors:** Vladimir A. Potapov, Roman S. Ishigeev, Svetlana V. Amosova

**Affiliations:** A. E. Favorsky Irkutsk Institute of Chemistry, Siberian Division of The Russian Academy of Sciences, 1 Favorsky Str., 664033 Irkutsk, Russia; ishigeev@irioch.irk.ru (R.S.I.); amosova@irioch.irk.ru (S.V.A.)

**Keywords:** 2*H*,3*H*-[1,4]thiazino[2,3,4-*ij*]quinolin-4-ium derivatives, annulation reactions, 8-quinolinesulfenyl halides, vinyl ethers, divinyl sulfide, divinyl selenide, phenyl vinyl sulfide, tetravinyl silane

## Abstract

Regioselective synthesis of novel 2*H*,3*H*-[1,4]thiazino[2,3,4-*ij*]quinolin-4-ium derivatives has been developed by annulation reactions of 8-quinolinesulfenyl halides with vinyl chalcogenides (vinyl ethers, divinyl sulfide, divinyl selenide and phenyl vinyl sulfide) and tetravinyl silane. The novel reagent 8-quinolinesulfenyl bromide was used in the annulation reactions. The influence of the substrate structure and the nature of heteroatoms on the direction of the reactions and on product yields has been studied. The opposite regiochemistry was observed in the reactions with vinyl chalcogenides and tetravinyl silane. The obtained condensed heterocycles are novel water-soluble functionalized compounds with promising biological activity.

## 1. Introduction

The quinoline ring continues to occupy an important place in medicinal chemistry as a valuable scaffold, presenting in many natural and biologically active compounds [1,2,3,4,5,6]. The quinoline scaffold is an integral part of a variety of drugs, including antimalarial medications (chloroquine, hydroxychloroquine, amodiaquine and primaquine) and fluoroquinolone antibiotics (ciprofloxacin, levofloxacin, gatifloxacin and moxifloxacin). Hydroxychloroquine in combination with other medications was recently used for the treatment of COVID-19 [7].

The sulfur-containing heterocycles are a structural part of many medications. Combinations of nitrogen heterocycles with condensed sulfur-containing rings have often played an important role in the discovery of new drugs [8]. Penicillin and cephalosporin scaffolds are examples of useful combinations of condensed nitrogen and sulfur-containing heterocycles in the structure of antibiotics.

**Figure 1:**
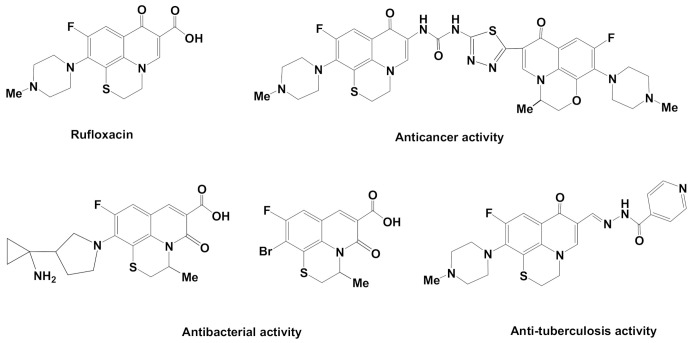
Known biologically active compounds containing 2*H*,3*H*-[1,4]thiazino[2,3,4-*ij*]quinolin-4-ium scaffold (anticancer [18], antibacterial [19] and antituberculosis [20] activity).

The combination of a quinoline heterocycle with a thiazine ring into a single fused molecule provides a very promising scaffold for medicinal chemistry [8,9,10,11]. The 2*H*,3*H*-[1,4]thiazino [2,3,4-*ij*]quinolin-4-ium derivatives represent an important family of compounds exhibiting various types of biological activity [12,13,14,15,16,17,18,19,20], including anticancer [18], antibacterial [19] and antituberculosis [20] action (Figure 1). The fluoroquinolone antibiotic rufloxacin is also a derivative of the 2*H*,3*H*-[1,4]thiazino[2,3,4-*ij*]quinolin-4-ium scaffold.

The laboratory of organochalcogen compounds of the A. E. Favorsky Irkutsk Institute of Chemistry develops effective approaches to novel chalcogen heterocycles by regioselective cyclization and annulation reactions of chalcogen-containing reagents [21,22,23,24,25,26].

Recently, we described the annulation reactions of 2-pyridinesulfenyl and 2-pyridineselenenyl halides with functionalized alkenes to afford 2*H*,3*H*-[1,3]thiazino[3,2-*a*]pyridin-4-ium and 2*H*,3*H*-[1,3]selenazolo[3,2-*a*]pyridin-4-ium derivatives in high yields [27,28,29,30,31,32].

In spite of some progress in synthetic methods for the preparation of 2*H*,3*H*-[1,4]thiazino[2,3,4-*ij*]quinolin-4-ium derivatives [33,34,35,36,37,38], the annulation reactions of 8-quinolinesulfenyl halides with vinylic heteroatom compounds, including vinylic ethers, sulfides, selenides and silanes, are hitherto unknown.

The creation of synthetic approaches to novel derivatives of the [1,4]thiazino[2,3,4-*ij*]quinolin-4-ium scaffold based on functionalized alkenes remains an important task. The goal of this research is the development of regioselective synthesis of novel condensed [1,4]thiazino[2,3,4-*ij*]quinolin-4-ium derivatives with promising biological activity based on the annulation reactions of 8-quinolinesulfenyl halides with vinylic heteroatom compounds (vinyl ethers, sulfides, selenides and silanes). Studying the influence of the substrate structure and the nature of the heteroatom in vinylic compounds on the direction of reactions and the yields of products is also a task of this research.

Efficient methods for the preparation of vinyl chalcogenides, including divinyl sulfide, divinyl selenide and divinyl telluride, have been previously developed in this institute [39,40,41,42,43,44,45].

## 2. Results and Discussion

**Scheme 1:**
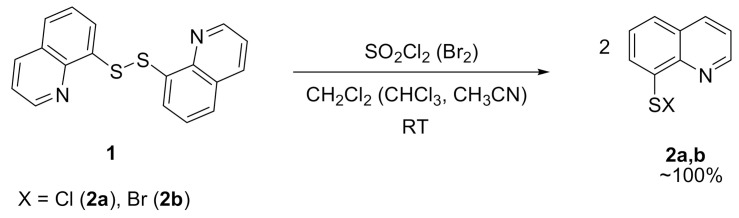
The generation of 8-quinolinesulfenyl halides **2a**, **b** from di(8-quinolinyl) disulfide **1** by the action of sulfuryl chloride or bromine.

Responding to the increasing demand for structurally diverse heterocyclic compounds with promising biological activity, we developed the synthesis of a new family of [1,4]thiazino[2,3,4-*ij*]quinolin-4-ium derivatives based on the annulation reactions of 8-quinolinesulfenyl chloride and bromide (**2a**,**b**) with vinylic heteroatom compounds (vinyl ethers, divinyl sulfide, divinyl selenide, phenyl vinyl sulfide and tetravinyl silane). Divinyl telluride was also studied in the reactions of sulfenyl halides **2a**, **b**. The latter compounds were generated in situ from di(8-quinolinyl) disulfide (**1**) by the action of sulfuryl chloride or bromine and used in further reactions without isolation (Scheme 1).

It should be emphasized that quinolinesulfenyl bromide **2b** was used in the reactions for the first time. There is no information about sulfenyl bromide **2b** in the literature (the SciFinder database).

**Scheme 2:**
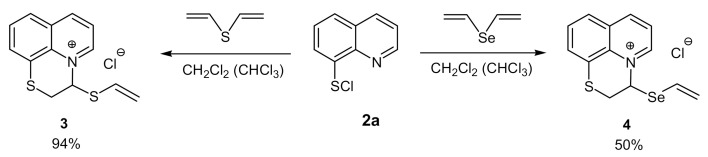
Synthesis of condensed compounds **3**, **4** from sulfenyl chloride **2a** and divinyl sulfide and selenide.

The reaction of sulfenyl chloride **2a** with divinyl sulfide and selenide resulted in the annulation of the dihydrothiazine cycle to the quinoline ring, affording water-soluble 3-(vinylsulfanyl)- and 3-(vinylselanyl)-2*H,*3*H*-[1,4]thiazino[2,3,4-*ij*]quinolin-4-ium chlorides **3**, **4** in 94% and 50% yields, respectively (Scheme 2).

The reaction of sulfenyl chloride **2a** with divinyl sulfide proceeded more easily than with divinyl selenide. The reaction with divinyl sulfide was carried out at room temperature for 24 h to give product **3** in 94% yield, whereas the process with divinyl selenide required 70 h in order to obtain product **4** in 50% yield. Attempts to intensify the reaction by refluxing the mixture of sulfenyl chloride **2a** and divinyl selenide led to an increase in the yield of compound **4** to 71%; however, the procedure was accompanied by the formation of some by-products, and in this case, the purification of the target compound **4** met with difficulties.

The presence of vinylsulfanyl and vinylselanyl groups in compounds **3** and **4** opens new possibilities for functionalization reactions as well as polymerization and copolymerization. It is known that vinyl sulfides can be easily involved in radical addition, polymerization and copolymerization due to their high reactivity in radical reactions [39]. For example, radical polymerization or oligomerization of compound **3** can afford water-soluble oligomeric or polymeric products, which may exhibit antibacterial activity.

**Scheme 3:**
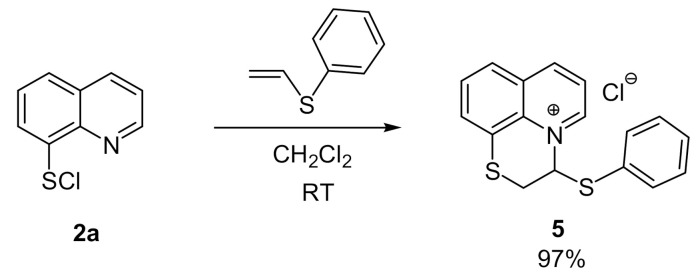
The reaction of sulfenyl chloride **2a** with phenyl vinyl sulfide.

Phenyl vinyl sulfide was involved in the reaction with sulfenyl chloride **2a**. The reaction was carried out at room temperature in methylene chloride to afford the condensed product, 3-(phenylsulfanyl)-2*H*,3*H*-[1,4]thiazino[2,3,4-*ij*]quinolin-4-ium chloride **5**, in 97% yield (Scheme 3). Like the reaction with divinyl sulfide, the annulation process proceeded in a regioselective mode, and the addition of the sulfur atom of sulfenyl chloride **2a** occurred exclusively at the β-position of the vinylsulfanyl group.

The reactions of sulfenyl bromide **2b** with divinyl sulfide, divinyl selenide and phenyl vinyl sulfide proceeded with low selectivity, and complex mixtures of products were obtained in each case.

**Scheme 4:**
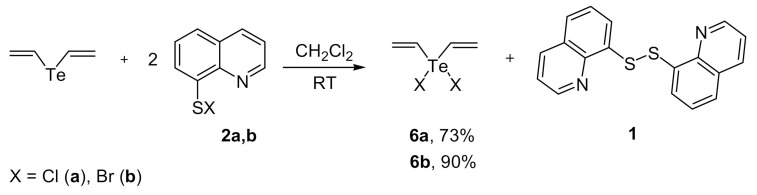
The formation of divinyltellurium dihalides **6a**, **b** in the reaction of sulfenyl halides **2a**, **b** with divinyl telluride.

The addition to the double bond was not observed in the reaction of sulfenyl halides **2a**, **b** with divinyl telluride. This reaction proceeded as halogenation of the tellurium atom with the formation of divinyltellurium dichloride and dibromide (**6a**, **b**). When a 2:1 ratio of sulfenyl halide **2a**, **b** and divinyl telluride was used, divinyltellurium dihalides **6a**, **b** were obtained in 73% and 90% yields, respectively (Scheme 4).

We suppose that the high affinity of the tellurium atom for halogens determines the course of this reaction. It is known that organic tellurides can be used as dehalogenation reagents. Previously, the formation of divinyltellurium dihalides **6a**, **b** in high yields was observed in the reactions of divinyl tellurides with bromine [46] as well as with selenium dichloride [47].

Tetravinyl silane was chosen as a representative of vinyl silanes, reactive compounds in the electrophilic addition due to the electron donating effect of the silicon atom, and studied in the reactions with sulfenyl halides **2a**, **b**. A 3-fold molar excess of tetravinyl silane with respect to sulfenyl halides **2a**, **b** was used in order to realize selective addition to only one vinyl group of tetravinyl silane.

**Scheme 5:**
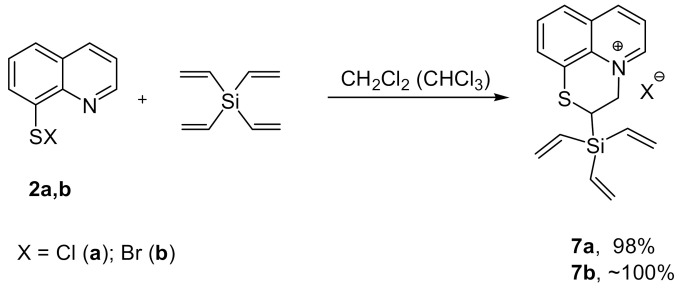
Synthesis of condensed compounds **7a**, **b** from sulfenyl halides **2a**, **b** and tetravinyl silane.

The reaction of sulfenyl halides **2a**, **b** with tetravinyl silane was found to proceed with opposite regiochemistry compared to the annulation with divinyl sulfide, divinyl selenide and phenyl vinyl sulfide. The products containing the sulfur atom attached to the α-carbon atom of the vinyl group, 2-trivinylsilyl-2*H*,3*H*-[1,4]thiazino[2,3,4-*ij*]quinolin-4-ium halides **7a** and **7b**, were obtained in 98% and quantitative yields, respectively (Scheme 5).

Vinyl ethers are among the most reactive compounds in electrophilic addition and are promising substrates for annulation reactions. From vinyl ethers, isobutyl vinyl and 2-chloroethyl vinyl ethers were available, and we studied these compounds in the reactions with sulfenyl halides **2a**, **b**.

**Scheme 6:**
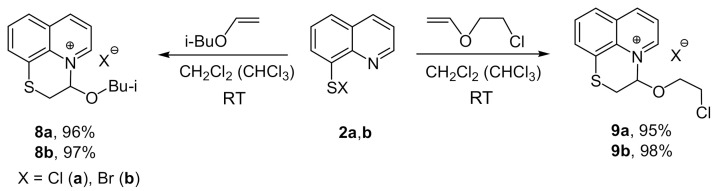
Synthesis of condensed products **8a**, **b** and **9a**, **b** from sulfenyl halides **2a**, **b** and isobutyl vinyl and 2-chloroethyl vinyl ethers.

The reactions smoothly proceeded at room temperature in methylene chloride or chloroform in a regioselective mode to afford the annulation products, 3-(2-methylpropoxy)- and 3-(2-chloroethyl)-*2H,3H*-[1,4]thiazino[2,3,4-*ij*]quinolin-4-ium chlorides (**8a**, **9a**) and bromides (**8b**, **9b**), in 95–98% yields (Scheme 6).

**Scheme 7:**
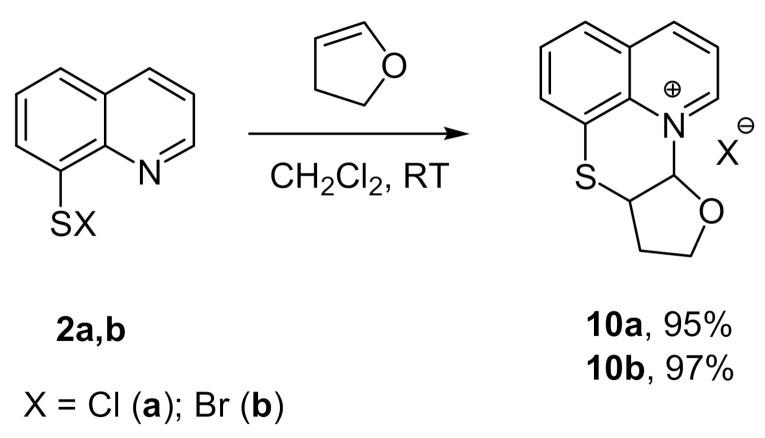
Synthesis of condensed products **10a**, **b** from sulfenyl halides **2a**, **b** and 2,3-dihydrofuran.

Cyclic vinyl ether, 2,3-dihydrofuran, was also involved in the reactions with sulfenyl halides **2a**, **b**. The reaction was carried out at room temperature in methylene chloride, leading to the condensed tetracyclic products, 7a*H*,8*H*,9*H*,10a*H*-furo[2′,3′:5,6]-2*H*,3*H*-[1,4]thiazino[2,3,4-*ij*]quinolin-11-ium halides **10a** and **10b**, in 95% and 97% yields, respectively (Scheme 7). Like the reaction with isobutyl vinyl and 2-chloroethyl vinyl ethers, the annulation process proceeded in a regioselective manner, giving only one isomer. The addition of the sulfur atom of sulfenyl halides **2a**, **b** occurred exclusively at the β-position of the vinyloxy moiety of 2,3-dihydrofuran.

**Scheme 8:**
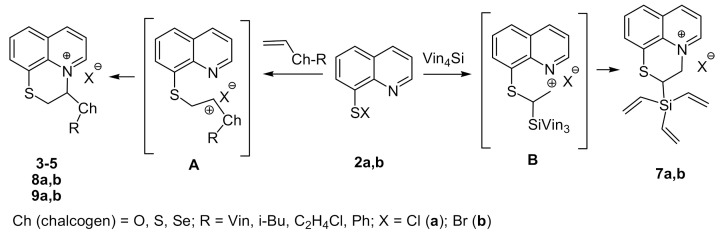
Directions of the reactions of sulfenyl halides **2a**, **b** with vinyl chalcogenides and tetravinyl silane.

Thus, the annulation reactions with vinyl chalcogen compounds (vinyl ethers, divinyl sulfide, divinyl selenide and phenyl vinyl sulfide) proceeded with the attachment of the sulfur atom of sulfenyl halides **2a**, **b** exclusively at the β-position of the vinylchacogenyl group (“Markovnikov direction”), whereas the addition of the sulfur atom occurred at the α-carbon atom of the vinylsilyl moiety in the case of tetravinyl silane (“anti-Markovnikov direction”). Possible intermediates **A** and **B** of these reactions (Scheme 8) can be considered to explain these trends.

It is known that the chalcogen atoms (oxygen, sulfur, selenium) stabilize well the adjacent carbocation as in intermediate **A**, which is formed by the addition of the sulfenyl cation at the β-position of the vinylchalcogenyl group (Scheme 8). On the other hand, it is known that electrophilic attack on the vinylsilyl moiety usually occurs at the α-carbon atom of the double bond, placing the developing carbocation at the β-position (β-effect, intermediate **B**) [48]. The β-effect of silicon is a special type of hyperconjugation that describes the stabilizing influence of the silicon atom on the development of positive charge at the β-carbon atom [49]. This effect manifests itself in the addition reaction of arenesulfenyl chlorides to vinyl silanes, leading to anti-Markovnikov adducts [48], whereas the reactions of arenesulfenyl chlorides with vinyl ethers and vinyl sulfides proceed with the opposite regiochemistry [50].

The structural assignments of synthesized compounds were made using ^1^H and ^13^C-NMR spectroscopy (Supplementary Materials containing examples of ^1^H- and ^13^C-NMR spectra are available online), including two-dimensional experiments, and were confirmed by elemental analysis. The products of opposite regiochemistry have characteristic signals of the carbon atoms bonded with charged nitrogen (N^+^): CH_2_N^+^ (~60 ppm) in ^13^C-NMR spectra of products **7a**, **b** (obtained from tetravinyl silane), SCHN^+^ (~73 and ~75 ppm) in ^13^C-NMR spectra of products **3**,**5** (obtained from vinyl sulfides) and OCHN^+^ (~93–97 ppm) in ^13^C-NMR spectra of products **8a**, **b–10a**, **b** (obtained from vinyl ethers and 2,3-dihydrofuran).

## 3. Experimental Section

### 3.1. General Information

^1^H (400.1 MHz), ^13^C (100.6 MHz) and ^29^Si (79.5 MHz) NMR spectra were recorded on a Bruker DPX-400 spectrometer (Bruker BioSpin GmbH, Rheinstetten, Germany) in 2–5% solution in D_2_O. ^1^H, ^13^C and ^29^Si chemical shifts (δ) are reported in parts per million (ppm), relative to tetramethylsilane (external) or to the residual solvent peaks of D_2_O (δ = 4.79). Elemental analysis was performed on a Thermo Scientific FLASH 2000 Organic Elemental Analyzer (Thermo Fisher Scientific Inc., Milan, Italy). Analytical determination of silicon was made by the gravimetric method [51]. Melting points were determined on a Kofler Hot-Stage Microscope PolyTherm A apparatus (Wagner & Munz GmbH, München, Germany). Absolute solvents were used in the reactions.

### 3.2. Synthesis of Compounds ***3–10a**,**b***

*3-(Vinylsulfanyl)-2H,3H-[1,4]thiazino[2,3,4-ij]quinolin-4-ium chloride* (**3**). A solution of sulfuryl chloride (0.079 g, 0.59 mmol) in chloroform (10 mL) was added dropwise to a solution of di(8-quinolinyl) disulfide (0.188 g, 0.59 mmol) in chloroform (10 mL), and the mixture was stirred for 10 min at room temperature. A solution of divinyl sulfide (0.101 g, 1.18 mmol) in chloroform (10 mL) was added dropwise, and the reaction mixture was stirred for 24 h at room temperature. The solvent was removed by rotary evaporator. The residue was washed with hexane and dried in vacuum, giving product **3** (0.311 g, 94% yield) as a light yellow oil. ^1^H-NMR (400 MHz, D_2_O): δ 3.86 (dd, *J* 14.4, 3.0 Hz, 1H, SCH_2_), 4.09 (dd, *J* 14.4, 2.3 Hz, 1H, SCH_2_), 5.39 (d, *J* 16.4 Hz, 1H, =CH_2_,), 5.68 (d, *J* 9.0 Hz, 1H, =CH_2_), 6.71 (s, 1H, NCH), 6.78 (dd, *J* 16.4, 9.0 Hz, 1H, SCH=), 7.88–7.92 (m, 1H, C_quino_), 8.01–8.04 (m, 1H, C_quino_), 8.11–8.16 (m, 2H, C_quino_), 9.13–9.17 (m, 2H, C_quino_). ^13^C-NMR (101 MHz, D_2_O): δ 28.95 (SCH_2_), 73.19 (NCH), 121.15 (=CH_2_), 125.47 (C_quino_), 126.47 (C_quino_), 127.96 (C_quino_), 129.57 (=CH), 131.62 (C_quino_), 131.80 (C_quino_), 133.49 (C_quino_), 134.35(C_quino_), 147.28 (C_quino_), 150.39 (C_quino_). Anal. Calcd for C_13_H_12_ClNS_2_): C 55.40, H 4.29, Cl 12.58, N 4.97, S 22.76. Found: C 55.65, H 4.53, Cl 12.29, N 5.15, S 23.01.

*3-(Vinylselanyl)-2H,3H-[1,4]thiazino[2,3,4-ij]quinolin-4-ium chloride* (**4**). A solution of sulfuryl chloride (0.095 g, 0.7 mmol) in chloroform (10 mL) was added dropwise to a solution of di(8-quinolinyl) disulfide (0.224 g, 0.7 mmol) in chloroform (10 mL), and the mixture was stirred for 10 min at room temperature. A solution of divinyl selenide (0.186 g, 1.4 mmol) in chloroform (10 mL) was added dropwise, and the reaction mixture was stirred for 70 h at room temperature. The solvent was removed by rotary evaporator. The residue was washed with hexane and dried in vacuum, giving product **4** (0.23 g, 50% yield) as a light yellow oil. ^1^H-NMR (400 MHz, D_2_O): δ 3.83 (dd, *J* 14.3, 3.2 Hz, 1H, SeCH_2_), 4.09 (dd, *J* 14.3, 2.3 Hz, 1H, SeCH_2_), 5.49 (d, *J* 16.7 Hz, 1H, =CH_2_), 5.99 (d, *J* 9.0 Hz, 1H, =CH_2_), 6.77 (m, 1H, NCH), 7.03 (dd, *J* 16.7, 9.0 Hz, 1H, SeCH=), 7.77–7.81 (m, 1H, C_quino_), 7.92–7.95 (m, 1H, C_quino_), 8.00–8.05 (m, 2H, C_quino_), 9.04–9.08 (m, 2H, C_quino_). ^13^C-NMR (101 MHz, D_2_O): δ 30.09 (SeCH_2_), 66.78 (NCH), 121.39 (=CH_2_), 121.56 (C_quino_), 125.50 (=CH), 128.06 (C_quino_), 129.65 (C_quino_), 129.95 (C_quino_), 130.29 (C_quino_), 131.79 (C_quino_), 133.59 (C_quino_), 146.91 (C_quino_), 150.12 (CN^+^,C_quino_). Anal. Calcd for C_13_H_12_ClNSSe: C 47.50, H 3.68, Cl 10.79, N 4.26, S 9.75. Found: C 47.35, H 3.83, Cl 10.61, N 4.45, S 10.02.

*3-(Phenylsulfanyl)-2H,3H-[1,4]thiazino[2,3,4-ij]quinolin-4-ium chloride* (**5**). A solution of sulfuryl chloride (0.081 g, 0.6 mmol) in methylene chloride (5 mL) was added dropwise to a solution of di(8-quinolinyl) disulfide (0.192 g, 0.6 mmol) in methylene chloride (10 mL), and the mixture was stirred for 10 min at room temperature. A solution of phenyl vinyl sulfide (0.163 g, 1.2 mmol) in methylene chloride (10 mL) was added dropwise, and the reaction mixture was stirred for 24 h at room temperature. The solvent was removed by rotary evaporator. The residue was washed with hexane and dried in vacuum, giving product **5** (0.386 g, 97% yield) as a light yellow oil. ^1^H-NMR (400 MHz, D_2_O): δ 3.92 (dd, *J* 14.4, 2.9 Hz, 1H, SCH_2_), 4.10 (dd, *J* 14.4, 2.0 Hz, 1H, SCH_2_), 6.52 (s, 1H, NCH), 7.09–7.11 (m, 2H, Ar), 7.33–7.37 (m, 2H, Ar), 7.50–7.57 (m, 2H, C_quino_), 7.85–7.89 (m, 1H, Ar), 8.07–8.10 (m, 3H, C_quino_), 9.02–9.04 (m, 1H, C_quino_). ^13^C-NMR (101 MHz, D_2_O): δ 28.24 (SCH_2_), 75.21 (NCH), 120.30 (Ar), 125.16 (C_quino_), 126.99 (C_quino_), 127.64 (C_quino_), 129.31 (C_quino_), 129.94 (Ar), 130.82 (C_quino_), 131.23 (Ar), 132.96 (C_quino_), 135.50 (Ar), 146.41 (C_quino_), 150.04 (C_quino_). Anal. Calcd for C_17_H_14_ClNS_2_: C 61.52, H 4.25, N 4.22, Cl 10.68, S 19.32. Found: C 61.94, H 4.52, N 4.42, Cl 10.93, S 19.13.

*2-(Trivinylsilyl)-2H,3H-[1,4]thiazino[2,3,4-ij]quinolin-4-ium chloride***(7a).** A solution of sulfuryl chloride (87 mg, 0.64 mmol) in methylene chloride (5 mL) was added dropwise to a solution of di(8-quinolinyl) disulfide (206 mg, 0.64 mmol) in methylene chloride (10 mL), and the mixture was stirred for 10 min at room temperature. The obtained solution of 8-quinolinesulfenyl chloride was added dropwise to a solution of tetravinylsilane (500 mg, 3.68 mmol) in methylene chloride (10 mL), and the reaction mixture was stirred for 72 h at room temperature. The solvent was removed by rotary evaporator. The residue was washed with hexane and dried in vacuum, giving product **7a** (416 mg, 98% yield) as a dark yellow oil. ^1^H-NMR (400 MHz, D_2_O): δ 3.42 (m, 1H, SCH), 5.07–5.11 (m, 1H, NCH_2_), 5.34 (m, 1H, NCH_2_), 6.04 (m, 3H, SiCH=), 6.20–6.35 (m, 6H, =CH_2_), 7.75–7.80 (m, 1H, C_quino_), 7.95–8.06 (m, 3H, C_quino_), 9.05–9.09 (m, 2H, C_quino_). ^13^C-NMR (101 MHz, D_2_O): δ 22.42 (SCH), 60.91 (NCH_2_), 121.60 (C_quino_), 127.18 (C_quino_), 129.30 (SiCH=CH_2_), 131.18 (C_quino_), 132.65 (C_quino_), 134.08 (C_quino_), 135.36 (C_quino_), 139.03 (SiCH=CH_2_), 148.38 (C_quino_), 149.12 (C_quino_), 182.44 (C_quino_). ^29^Si-NMR (400 MHz, D_2_O): δ –21.78. Anal. Calcd for C_17_H_18_ClNSSi: C 61.51, H 5.47, Cl 10.68, N 4.22, S 9.66, Si 8.46. Found: C 61.87; H 5.65; Cl 10.76, N 4.09, S 9.93, Si 8.25.

*2-(Trivinylsilyl)-2H,3H-[1,4]thiazino[2,3,4-ij]quinolin-4-ium bromide* (**7b**). A solution of bromine (45 mg, 0.28 mmol) in methylene chloride (2 mL) was added dropwise to a solution of di(8-quinolinyl) disulfide (90 mg, 0.28 mmol) in methylene chloride (10 mL), and the mixture was stirred for 10 min at room temperature. The obtained solution of 8-quinolinesulfenyl bromide was added dropwise to a solution of tetravinylsilane (230 mg, 1.69 mmol) in methylene chloride (10 mL), and the reaction mixture was stirred for 72 h at room temperature. The solvent was removed by rotary evaporator, and the residue was dried in vacuum, giving product **7b** (211 mg, quantitative yield) as a dark yellow oil. ^1^H-NMR (400 MHz, D_2_O): δ 3.46 (m, 1H, SCH), 5.12–5.19 (m, 1H, NCH_2_), 5.35 (m, 1H, NCH_2_), 6.02–6.08 (m, 3H, SiCH=), 6.21–6.34 (m, 6H, =CH_2_), 7.80–7.84 (m, 1H, C_quino_), 7.95–8.07 (m, 3H, C_quino_), 9.05–9.10 (m, 2H, C_quino_). ^13^C-NMR (101 MHz, D_2_O): δ 22.78 (SCH), 61.35 (NCH_2_), 121.94 (C_quino_), 127.58 (C_quino_), 129.79 (SiCH=CH_2_), 131.64 (C_quino_), 133.09 (C_quino_), 134.12 (C_quino_), 135.41 (C_quino_), 139.37 (SiCH=CH_2_), 148.76 (C_quino_), 149.51 (C_quino_), 188.91 (C_quino_). ^29^Si-NMR (400 MHz, D_2_O): δ –21.83. Anal. Calcd for C_17_H_18_BrNSSi: C 54.25, H 4.82, Br 21.23, N 3.72, S 8.52, Si 7.46. Found: C 54.67, H 5.01, Br 21.05, N 3.91, S 8.32, Si 7.28.

*3-(2-Methylpropoxy)-2H,3H-[1,4]thiazino[2,3,4-ij]quinolin-4-ium chloride* (**8a**)**.** A solution of sulfuryl chloride (46 mg, 0.34 mmol) in methylene chloride (2 mL) was added dropwise to a solution of di(8-quinolinyl) disulfide (109 mg, 0.34 mmol) in methylene chloride (8 mL), and the mixture was stirred for 10 min at room temperature. The obtained solution of 8-quinolinesulfenyl chloride was added dropwise to a solution of isobutyl vinyl ether (80 mg, 0.8 mmol) in methylene chloride (10 mL), and the reaction mixture was stirred for 20 h at room temperature. The solvent was removed by rotary evaporator. The residue was washed with hexane and dried in vacuum, giving product **8a** (193 mg, 96% yield) as a light yellow powder, mp 138–139 °C (decomp). ^1^H-NMR (400 MHz, D_2_O): δ 0.68 (d, *J* 6.7 Hz, 3H, CH_3_), 0.80 (d, *J* 6.7 Hz, 3H, CH_3_), 1.74–1.81 (m, 1H, CH), 3.29–3.33 (m, 1H, SCH_2_), 3.71–3.84 (m, 3H, OCH_2_, SCH_2_), 6.49 (m, 1H, NCH), 7.86–7.90 (m, 1H, C_quino_), 8.08–8.15 (m, 3H, C_quino_), 9.22–9.24 (m, 1H, C_quino_), 9.34–9.36 (m, 1H, C_quino_). ^13^C-NMR (101 MHz, D_2_O): δ 18.01 (CH_3_), 27.46 (CH), 28.48 (SCH_2_), 76.50 (OCH_2_), 91.67 (NCH), 121.13 (C_quino_), 122.41 (C_quino_), 127.43 (C_quino_), 129.49 (C_quino_), 133.11 (C_quino_), 134.24 (C_quino_), 148.12 (C_quino_), 149.11 (C_quino_), 151.09 (C_quino_). Anal. Calcd for C_15_H_18_ClNOS: C 60.90, H 6.13, Cl 11.98, N 4.73, S 10.84. Found: C 61.18, H 6.29, Cl 12.17, N 4.56, S 11.05.

*3-(2-Methylpropoxy)-2H,3H-[1,4]thiazino[2,3,4-ij]quinolin-4-ium bromide* (**8b**). A solution of bromine (42 mg, 0.26 mmol) in methylene chloride (2 mL) was added dropwise to a solution of di(8-quinolinyl) disulfide (84 mg, 0.26 mmol) in methylene chloride (8 mL), and the mixture was stirred for 10 min at room temperature. The obtained solution of 8-quinolinesulfenyl bromide was added dropwise to a solution of isobutyl vinyl ether (60 mg, 0.6 mmol) in methylene chloride (10 mL), and the reaction mixture was stirred for 20 h at room temperature. The solvent was removed by rotary evaporator. The residue was washed with hexane and dried in vacuum, giving product **8a** (172 mg, 97% yield) as a light yellow powder, mp 135–137 °C (decomp). ^1^H-NMR (400 MHz, D_2_O): δ 0.68 (d, *J* 6.6 Hz, 3H, CH_3_), 0.80 (d, *J* 6.6 Hz, 3H, CH_3_), 1.74–1.81 (m, 1H, CH), 3.31 (m, 1H, SCH_2_), 3.70–3.74 (m, 2H, OCH_2_), 3.82 (m, 1H, SCH_2_), 6.49 (m, 1H, NCH), 7.86–7.90 (m, 1H, C_quino_), 8.07–8.15 (m, 3H, C_quino_), 9.20–9.24 (m, 1H, C_quino_), 9.33–9.36 (m, 1H, C_quino_). ^13^C-NMR (101 MHz, D_2_O): δ 17.82, 17.88 (CH_3_), 27.32 (CH), 28.32 (SCH_2_), 76.35 (OCH_2_), 91.51 (NCH), 120.97 (C_quino_), 122.34 (C_quino_), 127.25 (C_quino_), 129.30 (C_quino_), 132.93 (C_quino_), 134.18 (C_quino_), 148.01 (C_quino_), 149.01 (C_quino_), 150.92 (C_quino_). Anal. Calcd for C_15_H_18_NBrOS: C 52.94, H 5.33, Br 23.48, N 4.12, S 9.42. Found: C 53.11, H 5.51, Br 23.65, N 3.98, S 9.23.

*3-(2-Chloroethoxy)-2H,3H-[1,4]thiazino[2,3,4-ij]quinolin-4-ium chloride***(9a)** was obtained as a light yellow oil in 95% yield under similar conditions to the synthesis of compound **8a**. ^1^H-NMR (400 MHz, D_2_O): δ 3.66–3.70 (m, 2H, CH_2_Cl), 3.78–3.85 (m, 2H, SCH_2_), 3.90–3.95 (m, 1H, OCH_2_), 4.27–4.32 (m, 1H, OCH_2_), 6.50 (d, *J* 1.5 Hz, 1H, NCH), 7.86–7.90 (m, 1H, C_quino_), 8.09–8.15 (m, 3H, C_quino_), 9.22–9.24 (m, 1H, C_quino_), 9.39–9.40 (m, 1H, C_quino_). ^13^C-NMR (101 MHz, D_2_O): δ 28.30 (SCH_2_), 42.60 (CH_2_Cl), 70.06 (OCH_2_), 91.39 (NCH), 121.30 (C_quino_), 125.55 (C_quino_), 127.55 (C_quino_), 129.53 (C_quino_), 131.68 (C_quino_), 133.26 (C_quino_), 148.29 (C_quino_), 151.33 (C_quino_). Anal. Calcd for C_13_H_13_NCl_2_OS: C 51.66, H 4.34, N 4.63, Cl 23.46, S 10.61. Found: C 51.95, H 4.66, N 4.89, Cl 23.31, S 10.39.

*3-(2-Chloroethoxy)-2H,3H-[1,4]thiazino[2,3,4-ij]quinolin-4-ium bromide***(9b)** was obtained as a light yellow oil in 98% yield under similar conditions to the synthesis of compound **8b**. ^1^H-NMR (400 MHz, D_2_O): δ 3.64–3.69 (m, 2H, CH_2_Cl), 3.76–3.84 (m, 2H, SCH_2_), 3.89–3.95 (m, 1H, OCH_2_), 4.25–4.31 (m, 1H, OCH_2_), 6.49 (d, *J* 1.5 Hz, 1H, NCH), 7.85–7.91 (m, 1H, C_quino_), 8.09–8.17 (m, 3H, C_quino_), 9.23–9.26 (m, 1H, C_quino_), 9.41–9.46 (m, 1H, C_quino_). ^13^C-NMR (101 MHz, D_2_O): δ 28.38 (SCH_2_), 42.67 (CH_2_Cl), 70.13 (OCH_2_), 91.42 (NCH), 121.35 (C_quino_), 125.59 (C_quino_), 127.60 (C_quino_), 129.57 (C_quino_), 131.76 (C_quino_), 133.32 (C_quino_), 148.33 (C_quino_), 151.42 (C_quino_). Anal. Calcd for C_13_H_13_BrClNOS: C 45.04, H 3.78, N 4.04, S 9.25. Found: C 44.79, H 3.96, N 3.98, S 9.45.

*7aH,8H,9H,10aH-Furo[2′,3′:5,6]-2H,3H-[1,4]thiazino[2,3,4-ij]quinolin-11-ium chloride***(10a).** A solution of sulfuryl chloride (60 mg, 0.44 mmol) in methylene chloride (5 mL) was added dropwise to a solution of di(8-quinolinyl) disulfide (141 mg, 0.44 mmol) in methylene chloride (10 mL), and the mixture was stirred for 10 min at room temperature. The obtained solution of 8-quinolinesulfenyl chloride was added dropwise to a solution of 2,3-dihydrofuran (63 mg, 0.9 mmol) in methylene chloride (10 mL), and the reaction mixture was stirred for 24 h at room temperature. The solvent was removed by rotary evaporator. The residue was washed with hexane and dried in vacuum, giving product **10a** (222 mg, 95% yield) as a light yellow oil. ^1^H-NMR (400 MHz, D_2_O): δ 1.90–2.01 (m, 1H, CH_2_), 2.62–2.70 (m, 1H, CH_2_), 4.14–4.19 (m, 1H, SCH), 4.31–4.35 (m, 1H, CH_2_O), 6.51 (d, 1H, OCHN^+^, ^3^*J* = 4.6 Hz), 7.86–7.90 (m, 1H, C_quino_), 8.12–8.18 (m, 3H, C_quino_), 9.16–9.18 (m, 1H, C_quino_), 9.57–9.59 (m, 1H, N^+^C_quino_). ^13^C-NMR (101 MHz, D_2_O): δ 28.08 (CH_2_), 37.55 (SCH), 69.30 (CH_2_O), 91.63 (OCHN^+^), 121.41 (C_quino_), 121.86 (C_quino_), 127.60 (C_quino_), 129.15 (C_quino_), 129.19 (C_quino_), 130.60 (C_quino_), 133.76 (C_quino_), 146.08 (C_quino_), 149.45 (CN^+^,C_quino_). Anal. Calcd for C_13_H_12_ClNOS: C 58.75, H 4.55, Cl 13.34, N 5.27, S 12.07. Found: C 59.02, H 4.38, Cl 13.61, N 5.47, S 11.93.

*7aH,8H,9H,10aH-Furo[2′,3′:5,6]-2H,3H-[1,4]thiazino[2,3,4-ij]quinolin-11-ium bromide***(10b).** A solution of bromine (70 mg, 0.44 mmol) in methylene chloride (10 mL) was added dropwise to a solution of di(8-quinolinyl) disulfide (141 mg, 0.44 mmol) in methylene chloride (10 mL), and the mixture was stirred for 10 min at room temperature. The obtained solution of 8-quinolinesulfenyl bromide was added dropwise to a solution of 2,3-dihydrofuran (63 mg, 0.9 mmol) in methylene chloride (10 mL), and the reaction mixture was stirred for 20 h at room temperature. The solvent was removed by rotary evaporator. The residue was washed with hexane and dried in vacuum, giving product **10b** (265 mg, 97% yield) as a light yellow oil. ^1^H-NMR (400 MHz, D_2_O): δ 1.93–2.04 (m, 1H, CH_2_), 2.65–2.74 (m, 1H, CH_2_), 4.14–4.19 (m, 1H, SCH), 4.29–4.35 (m, 1H, CH_2_O), 6.50 (d, 1H, OCHN^+^, ^3^*J* = 4.6 Hz), 7.84–7.88 (m, 1H, C_quino_), 8.09–8.18 (m, 3H, C_quino_), 9.15–9.17 (m, 1H, C_quino_), 9.54–9.57 m (1H, N^+^C_quino_). ^13^C-NMR (101 MHz, D_2_O): δ 27.83 (CH_2_), 37.29 (SCH), 69.05 (CH_2_O), 91.35 (OCHN^+^), 121.60 (C_quino_), 127.34 (C_quino_), 128.88 (C_quino_), 130.35 (C_quino_), 133.50 (C_quino_), 145.80 (C_quino_), 149.18 (CN^+^, C_quino_). Anal. Calcd for C_13_H_12_BrNOS: C 50.33, H 3.90, N 4.52, Br 25.76, S 10.34. Found: C 50.61, H 4.07, N 4.35, Br 26.03, S 10.53. 

## 4. Conclusions

An efficient synthetic approach to 2*H*,3*H*-[1,4]thiazino[2,3,4-ij]quinolin-4-ium derivatives has been developed by regioselective annulation reactions of 8-quinolinesulfenyl halides with vinyl chalcogenides (vinyl ethers, divinyl sulfide, divinyl selenide and phenyl vinyl sulfide) and tetravinyl silane. 8-Quinolinesulfenyl bromide was used for the first time. The obtained compounds **3**–**5**, **7a**, **b**–**10a**, **b** are water-soluble, and this property is favorable with respect to possible biological activeity.

The annulation reactions with vinyl chalcogenides proceeded with the attachment of the sulfur atom of 8-quinolinesulfenyl halides exclusively at the β-position of the vinylchacogenyl group, whereas the opposite regiochemistry was observed in the case of tetravinyl silane. The reactivity of vinyl chalcogenides in the annulation reactions was found to decrease in the following order: vinyl ethers > vinyl sulfides > vinyl selenide. This corresponds to the reactivity order of vinyl chalcogenides in electrophilic addition reactions [52].

## Data Availability

Not applicable.

## Supplementary Materials

The following are available online: examples of ^1^H- and ^13^C-NMR spectra of the obtained compounds.

## Sample Availability

Samples of the compounds are not available from the authors.
