# Supplementary material for: Efficient Regioselective Synthesis of Novel Water-Soluble 2H,3H-[1,4]thiazino[2,3,4-ij]quinolin-4-ium Derivatives by Annulation Reactions of 8-quinolinesulfenyl Halides
Source: Molecules. 2021 Feb 20;26(4):1116. doi: 10.3390/molecules26041116 (PMC7923262; doi:10.3390/molecules26041116)
Supplement: Supplementary file 1 [file molecules-26-01116-s001.pdf]

Supplementary Materials

Efficient regioselective synthesis of novel water-soluble 2***H***,3***H***-[1,4]thiazino[2,3,4-ij]quinolin-4-ium derivatives by annulation reactions of 8-quinolinesulfenyl halides

Vladimir A. Potapov*, Roman S. Ishigeev and Svetlana V. Amosova

A. E. Favorsky Irkutsk Institute of Chemistry, Siberian Division of The Russian Academy of Sciences, 1 Favorsky Str., Irkutsk 664033, Russia; ishigeev@irioch.irk.ru (R.S.I.); amosova@irioch.irk.ru (S.V.A.)
* Correspondence: v.a.potapov@mail.ru

Table of Contents

Experimental (General Information) **2**

Examples of ^{1}H-NMR Spectra **3-7**

Examples of ^{13}C-NMR Spectra **8-10**

Example of ^{29}Si-NMR Spectrum **10**

Experimental (General Information)

^{1}H (400.1 MHz), ^{13}C (100.6 MHz) and ^{29}Si (79.5 MHz) NMR spectra were recorded on a Bruker DPX-400 spectrometer (Bruker BioSpin GmbH, Rheinstetten, Germany) in 2-5% solution in D_2O. ^{1}H and ^{13}C chemical shifts (δ) are reported in parts per million (ppm), relative to tetramethylsilane (external) or to the residual solvent peaks of D_2O (δ = 4.79). Elemental analysis was performed on a Thermo Scientific FLASH 2000 Organic Elemental Analyzer (Thermo Fisher Scientific Inc., Milan, Italy). Melting points were determined on a Kofler Hot-Stage Microscope PolyTherm A apparatus (Wagner & Munz GmbH, München, Germany).

Examples of ^{1}H-NMR spectra

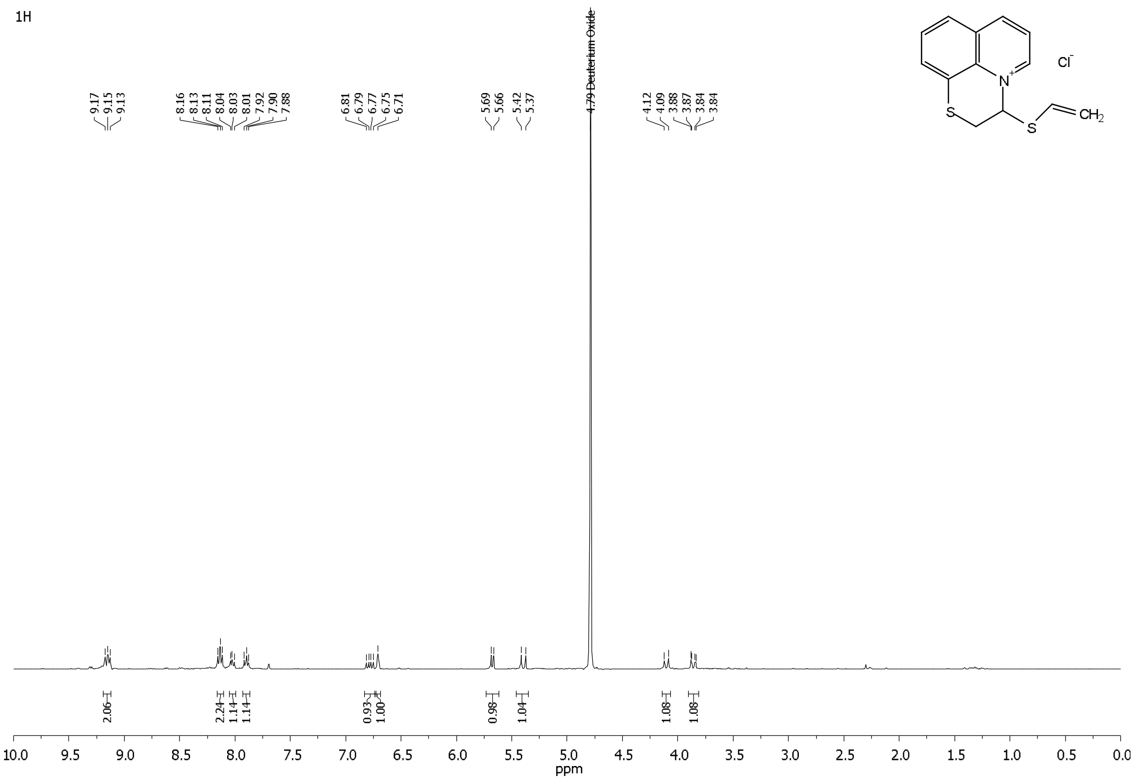

^{1}H-NMR (D_2O) spectrum of compound 3

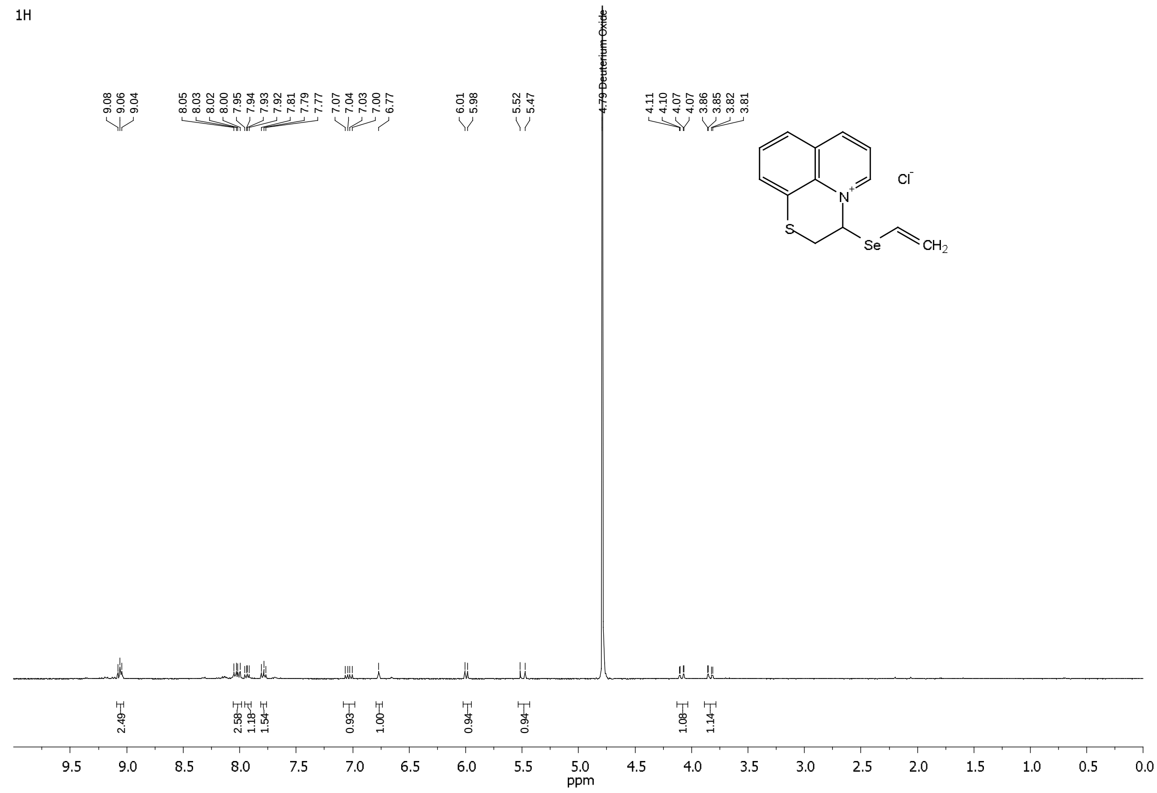

^{1}H-NMR (D_2O) spectrum of compound 4

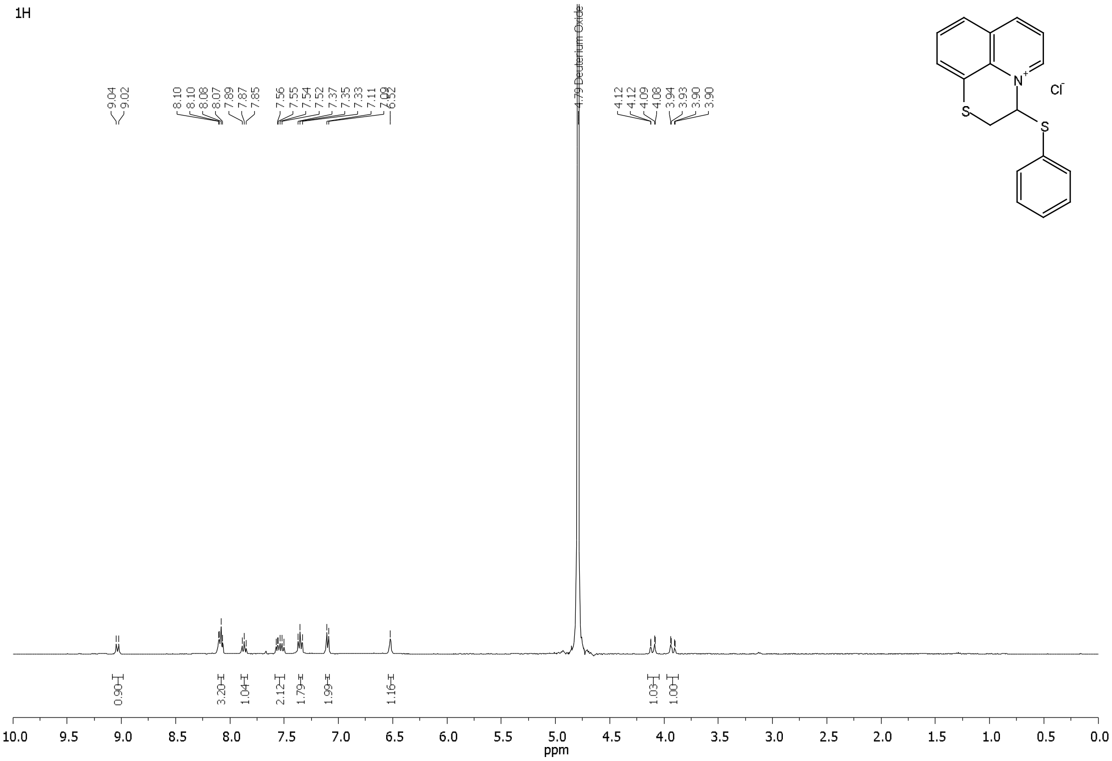

^{1}H-NMR (D_2O) spectrum of compound 5

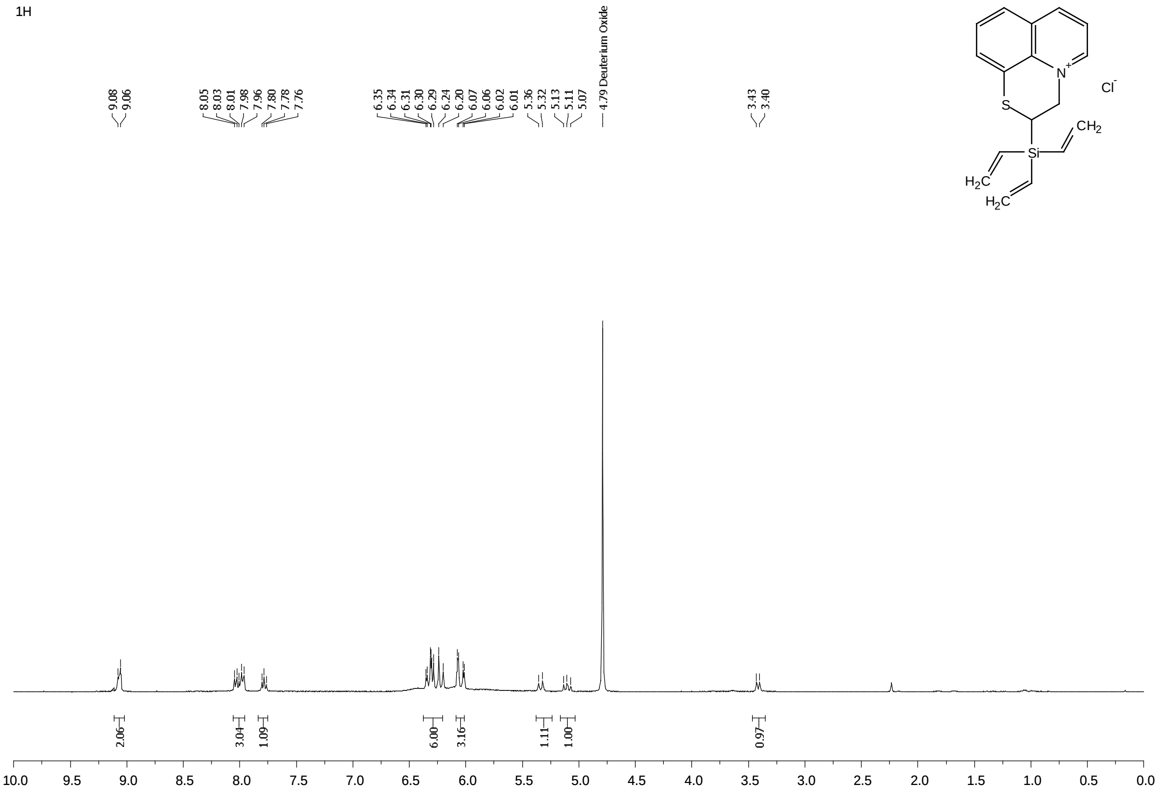

^{1}H-NMR (D_2O) spectrum of compound 7a

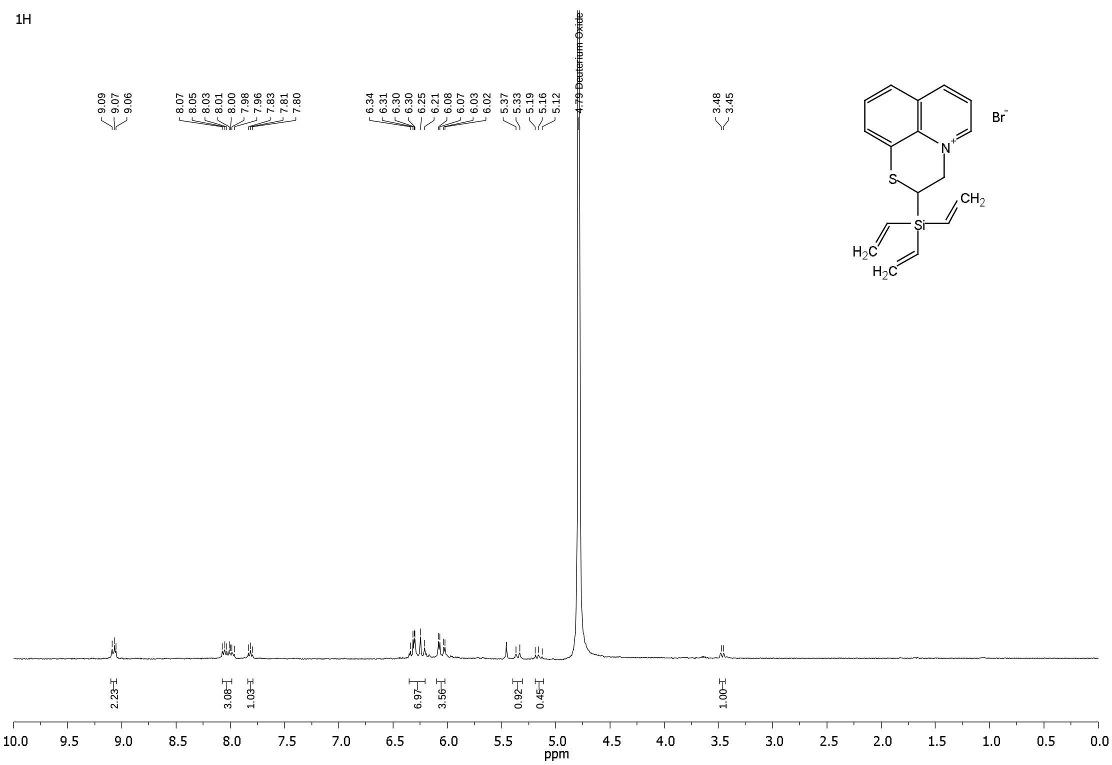

^{1}H-NMR (D_2O) spectrum of compound 7b

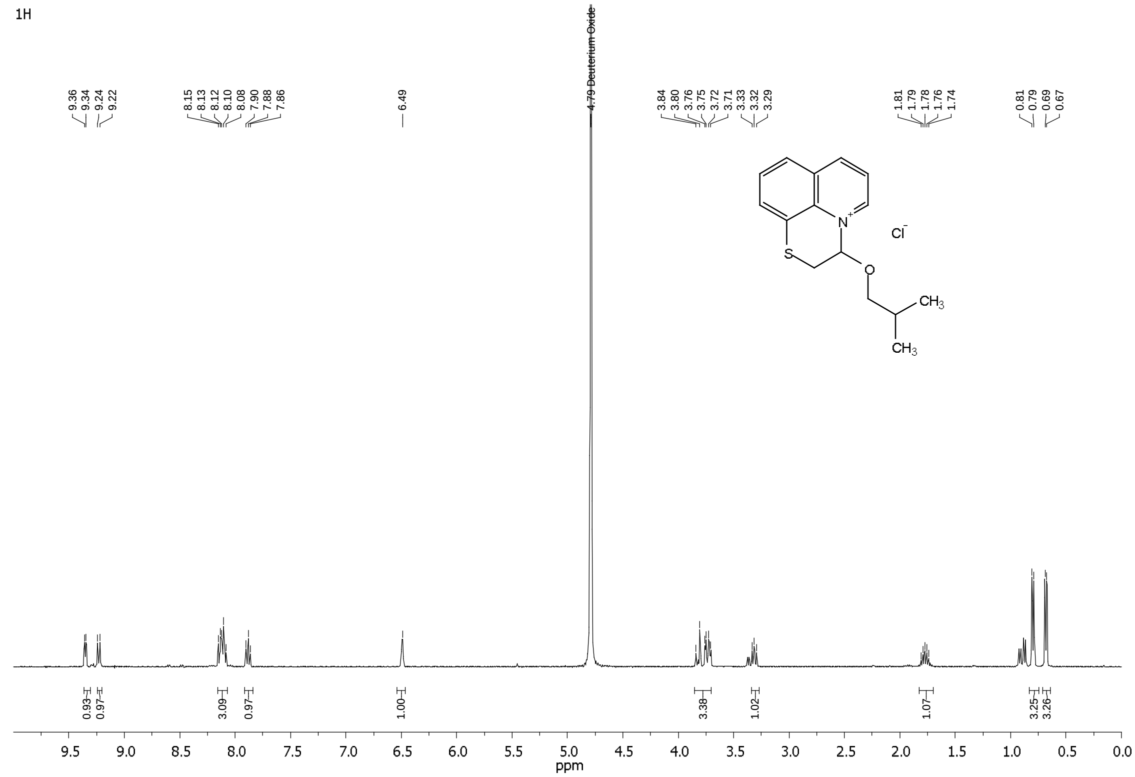

^{1}H-NMR (D_2O) spectrum of compound 8a

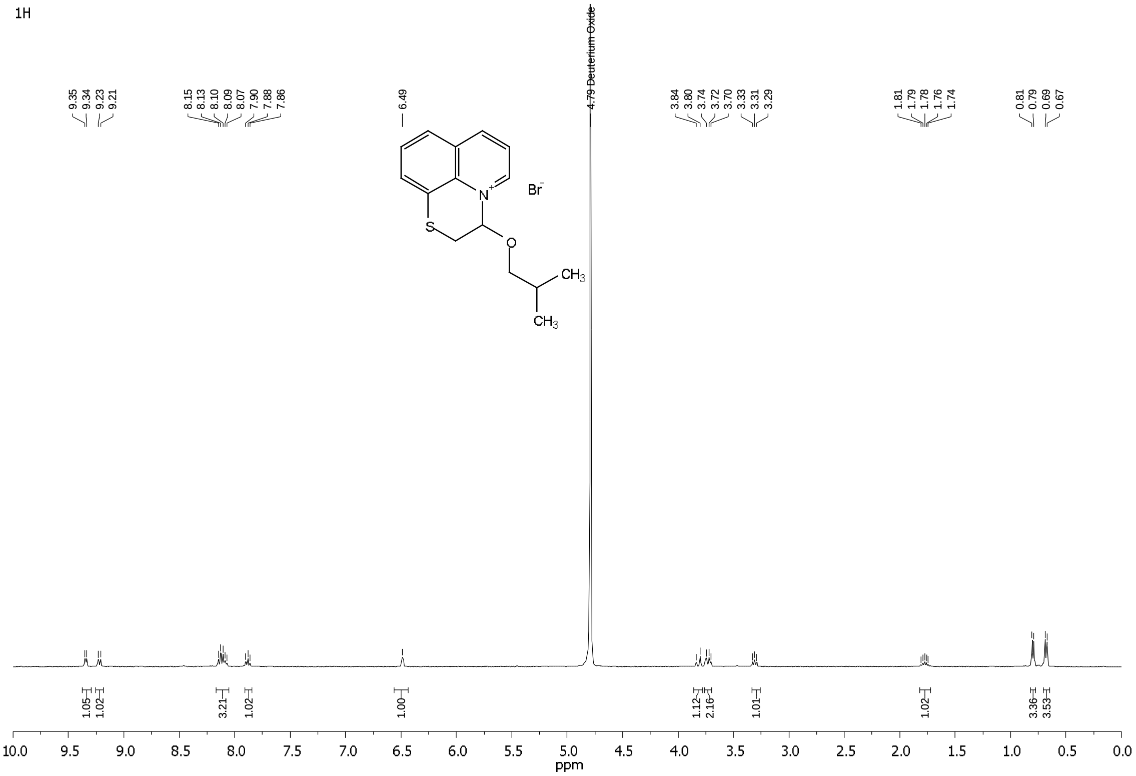

^{1}H-NMR (D_2O) spectrum of compound 8b

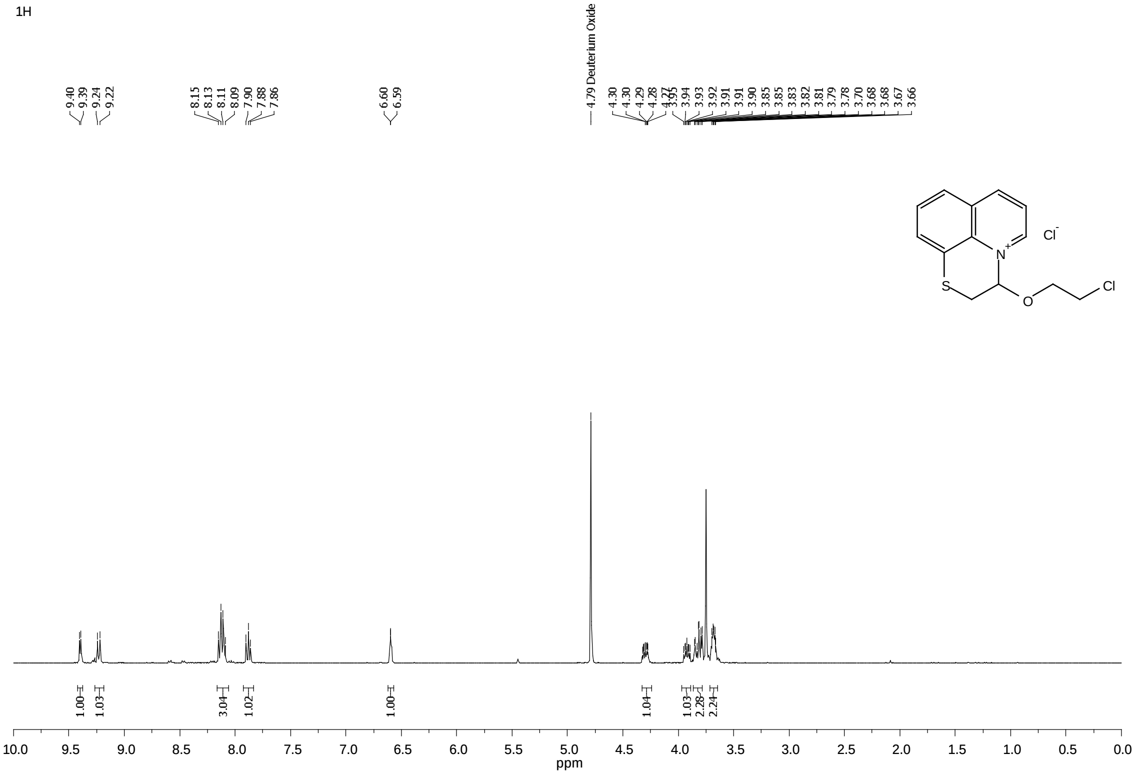

^{1}H-NMR (D_2O) spectrum of compound 9a

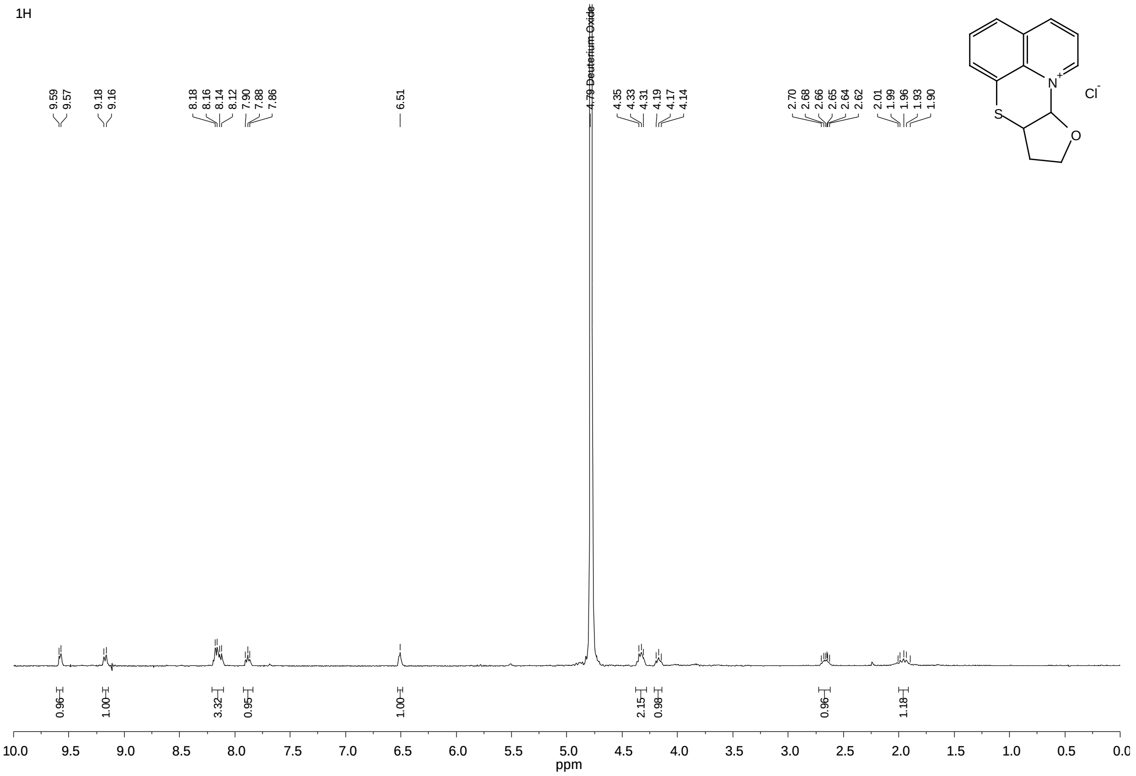

^{1}**H-NMR (D_2O) spectrum of compound 10a**

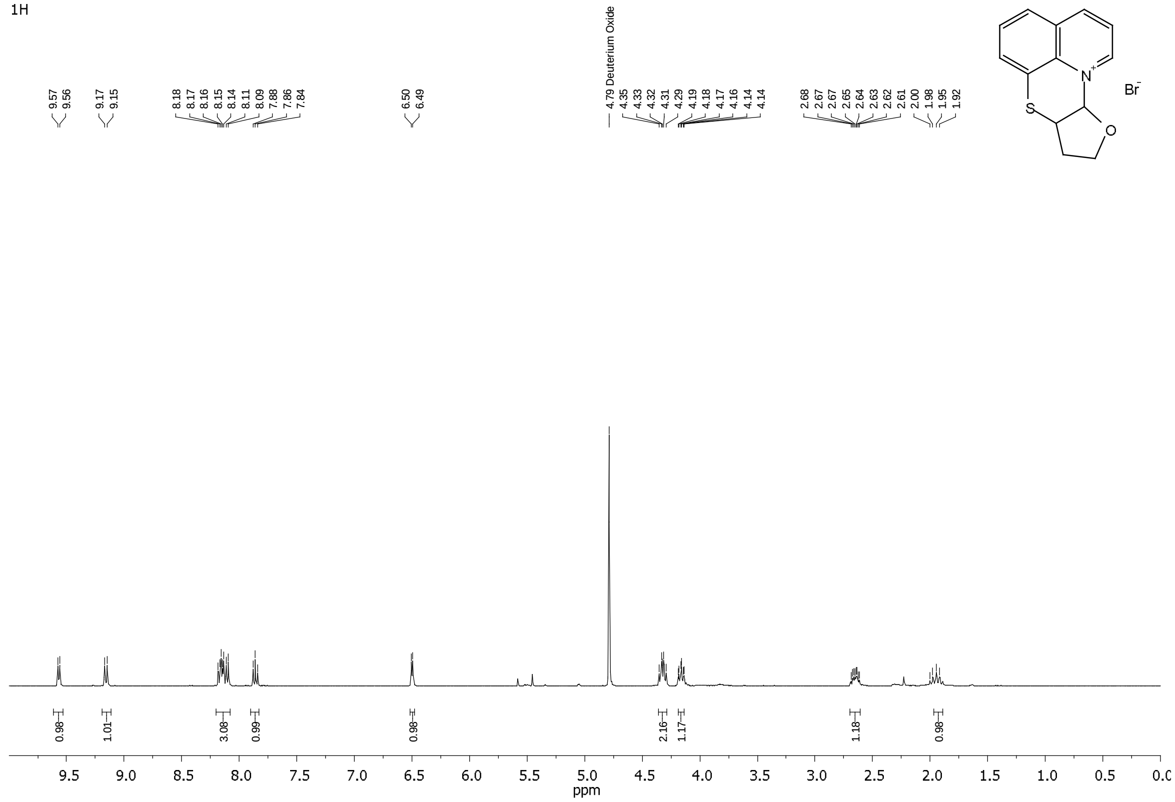

^{1}**H-NMR (D_2O) spectrum of compound 10b**

Examples of ^{13}C-NMR spectra

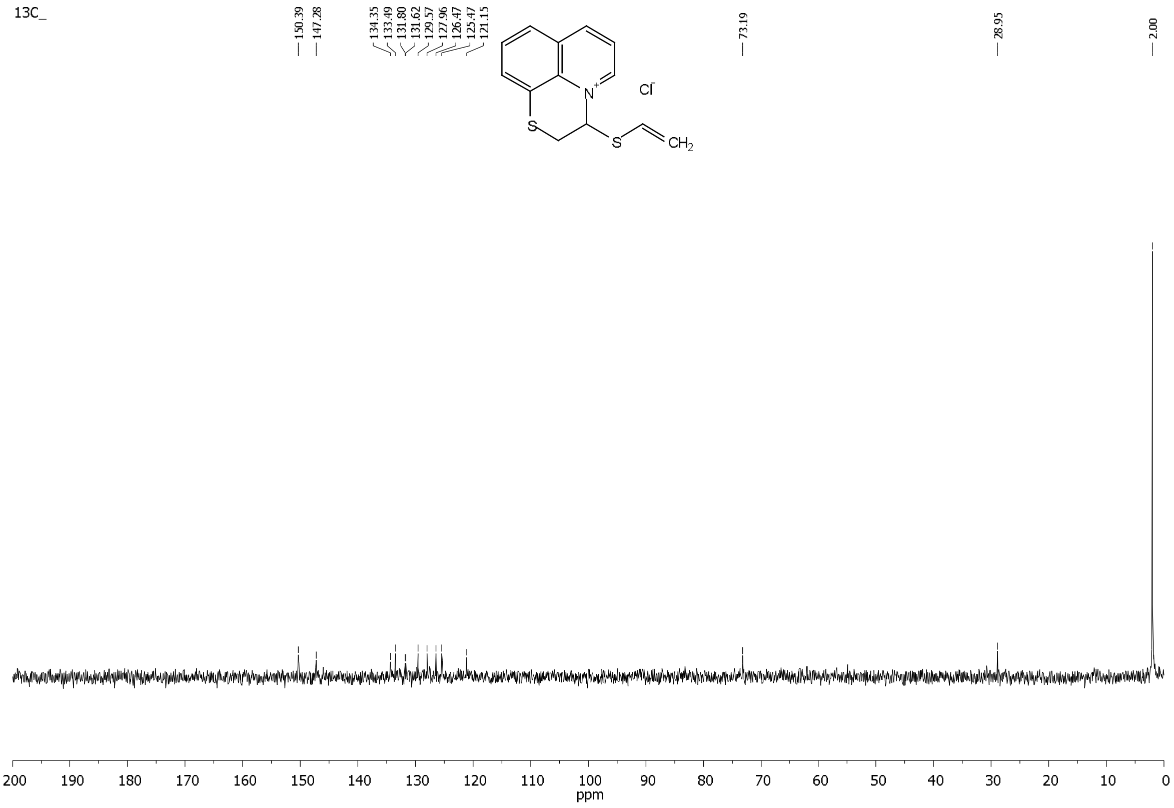

^{13}C-NMR (D_2O) spectrum of compound 3
($Me_3SiOSiMe_3$ as an external standard, 2 ppm)

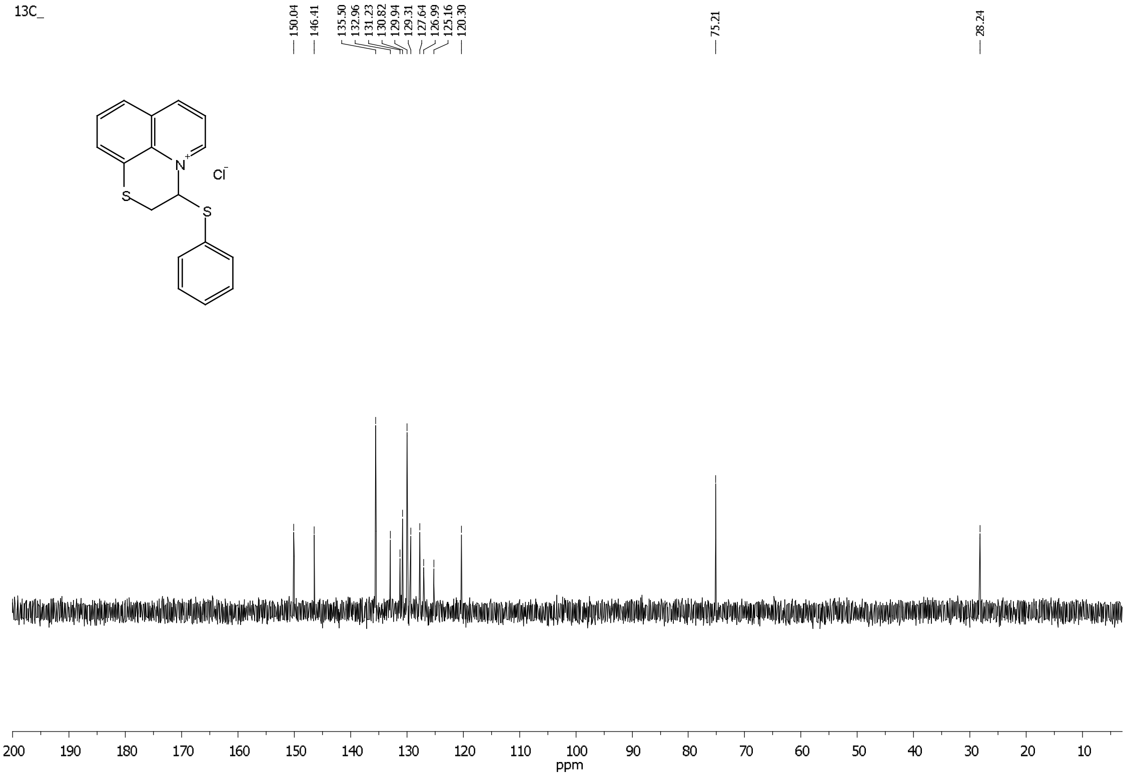

^{13}C-NMR (D_2O) spectrum of compound 5

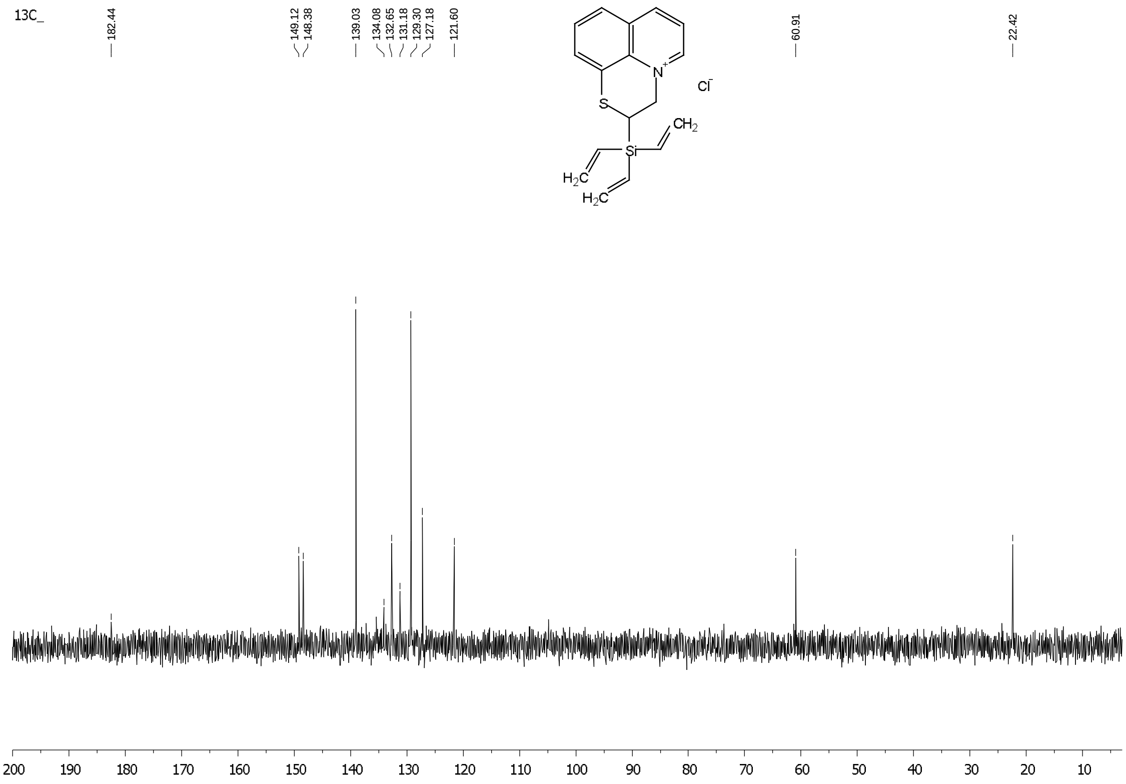

^{13}C-NMR (D_2O) spectrum of compound 7a

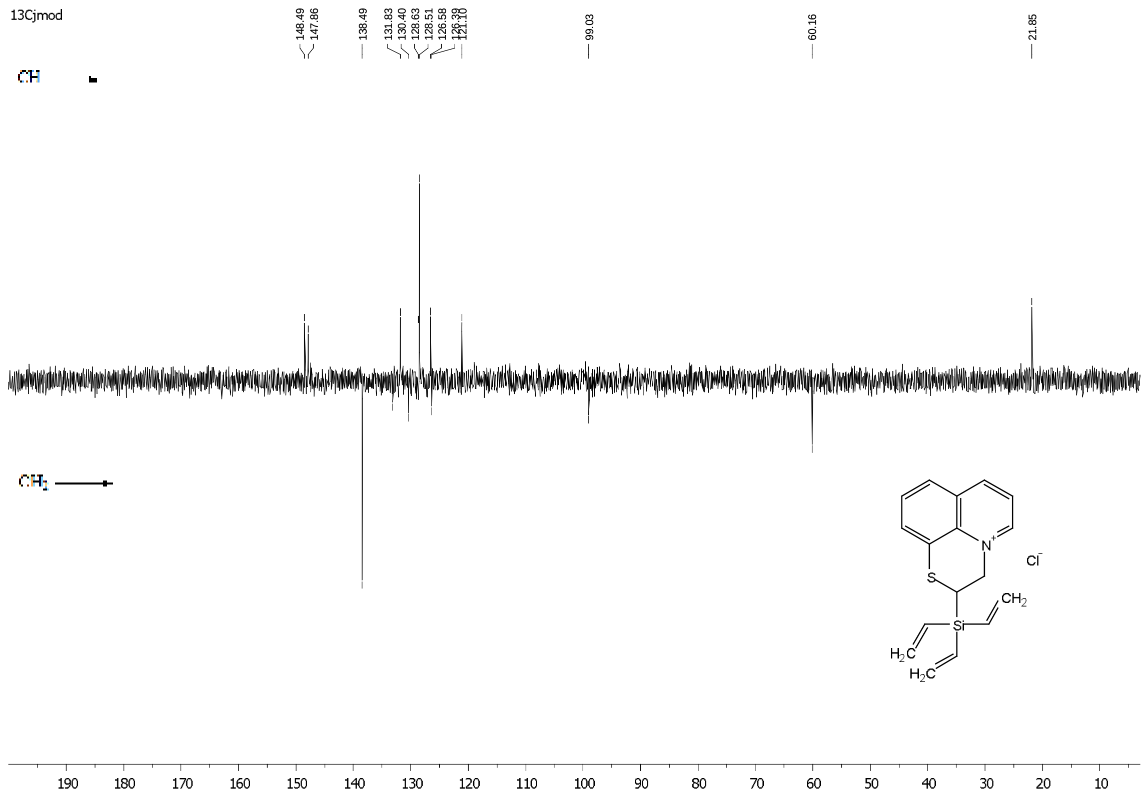

^{13}C-NMR J_{mod} (D_2O) spectrum of compound 7a

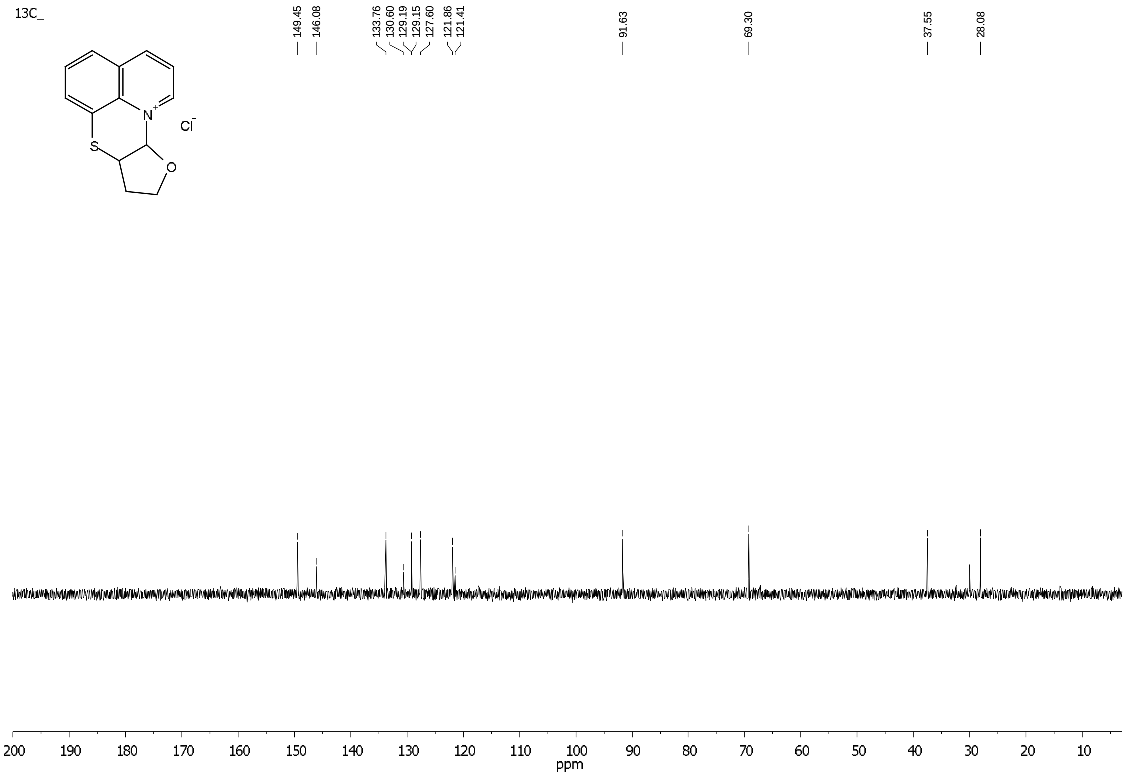

^{13}C-NMR (D_2O) spectrum of compound 10a

Example of ^{29}Si spectrum

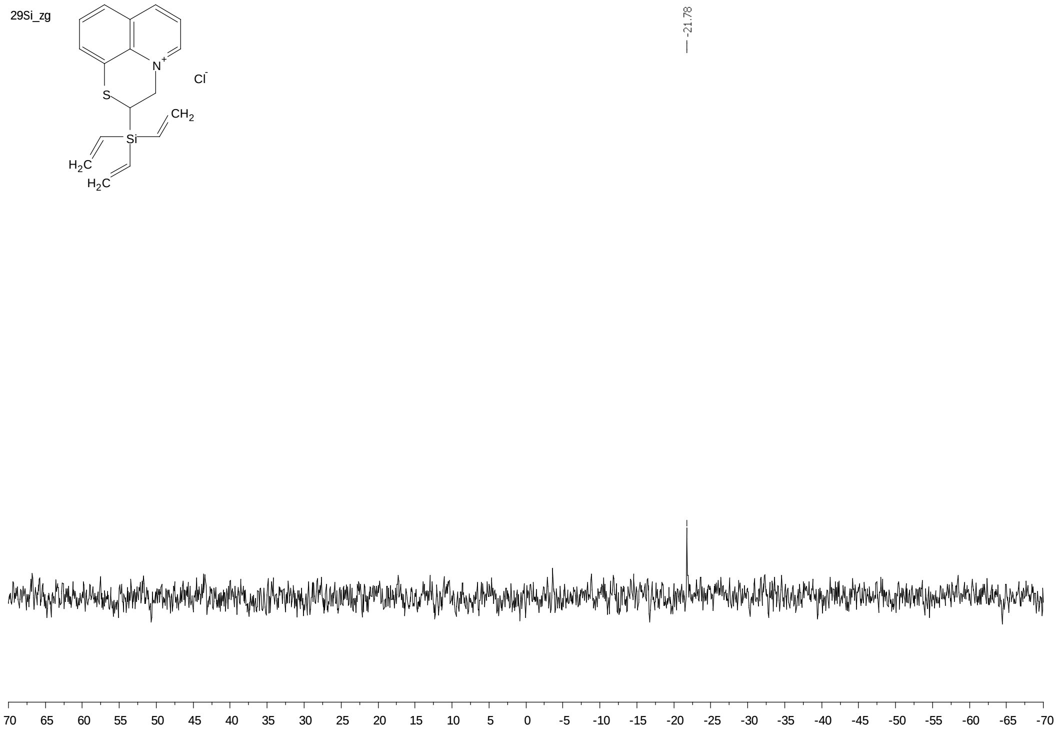

^{29}Si-NMR (D_2O) spectrum of compound 7a
